# Alternate serotype adenovector provides long-term therapeutic gene expression in the eye

**Published:** 2008-12-30

**Authors:** Melissa M. Hamilton, Gordon A. Byrnes, Jason G. Gall, Douglas E. Brough, C. Richter King, Lisa L. Wei

**Affiliations:** 1 Pre-Clinical Sciences, GenVec, Inc., Gaithersburg, MD; 2 Vector Core, GenVec, Inc., Gaithersburg, MD; 3 Vector Sciences, GenVec, Inc., Gaithersburg, MD; 4 Research, GenVec, Inc., Gaithersburg, MD; 5 Uniformed Services University of the Health Sciences, Bethesda, MD

## Abstract

**Purpose:**

To determine whether the duration of transgene expression from an alternate adenovector serotype, Ad35, can provide advantages over an Ad5 serotype vector following a single intravitreal (IVT) administration.

**Methods:**

To assess the transgene expression profile, mice received one IVT injection of Ad5- or Ad35-based vectors expressing green fluorescent protein (GFP), luciferase or pigment epithelium-derived factor (PEDF). At specified time points following vector administration, eyes were monitored for GFP expression, or eyes were harvested and assayed for adenovector genomes, luciferase activity or PEDF levels. Ad35-based vector in vivo biologic activity was investigated using a mouse model of laser-induced choroidal neovascularization (CNV). On Day 0, mice received one IVT injection of Ad5.PEDF or Ad35.PEDF (HI-RGD) followed by laser-induced CNV on Day 28. Fourteen days later, animals were perfused with fluorescein-labeled dextran and CNV lesion size quantitated in choroidal flat mounts.

**Results:**

These studies demonstrate that following a single IVT adenovector administration: 1) gene expression is prolonged following administration of an Ad35 compared to an Ad5-based vector; 2) the amount of vector genomes in the eye remain constant out to 60 days post injection of both Ad5 and Ad35-based vectors; and 3) an Ad35.PEDF (HI-RGD) vector inhibits CNV in a mouse model at 42 days post injection.

**Conclusions:**

These studies show that transgene and genome levels are prolonged in the eye following 1 IVT injection of an Ad35-based vector. Moreover, therapeutic gene levels from 1 IVT administration of Ad35.PEDF (HI-RGD) vector block abnormal blood vessel growth in a laser-induced CNV mouse model.

## Introduction

Neovascularization (NV) is the primary cause of blindness in a wide range of ocular diseases, such as diabetic retinopathy (DR) and age-related macular degeneration (AMD). Collectively, AMD and DR are the leading causes of permanent blindness in the developed world [1–6]. Currently in the United States, approximately 5.3 million individuals have DR and an estimated 1.75 million people have wet AMD [6–8]. These numbers are expected to increase with the rise in obesity and as more of our population becomes elderly [6,8,9]. Since AMD and diabetes are chronic diseases, long-term expression of a therapeutic product is likely to be required.

Recently substantial progress has been made toward the treatment of wet AMD with Phase III clinical trials using two different agents, Macugen^®^ and Lucentis^®^. Both have demonstrated that an anti-vascular endothelial growth factor (VEGF) strategy can delay disease progression and in the case of Lucentis^®^, improve vision for patients with wet AMD [10–13]. Both anti-VEGF approaches, however, require repeated intravitreal (IVT) injections at approximately 4–6 week intervals. Although these approaches are encouraging, the necessity for frequent ocular injections at such close intervals can be a cause of concern for both the patient and physician. One reason for this apprehension is the development of endophthalmitis (intraocular infection), which can require ocular surgery and lead to vision loss [14,15]. Another concern for both the patient and physician is the need for frequent physician visits by a predominately elderly patient population? Therefore, an important emphasis for ophthalmologic research is to reduce the number of ocular injections and develop less invasive procedures for drug delivery. Because of the high frequency of intraocular injections needed with Lucentis^®^ and Macugen^®^, we sought to identify methods that would reduce the number of intraocular injections. We have identified an alternative adenoviral vector serotype, Ad35, which provides prolonged gene expression, thereby offering longer-term activity with fewer IVT administrations.

Adenovectors are a useful protein expression system and have application for the treatment of chronic diseases, such as AMD [16]. Adenoviral vectors efficiently transduce many ocular cell types. Following IVT administration, several cell types in the anterior segment are transduced, including the iris and ciliary body epithelium, corneal endothelium, and trabecular meshwork [17–21]. In the posterior segment, retinal pigment epithelial, photoreceptor, and Müller cells are transduced [17–21]. Furthermore, adenovectors are well tolerated following IVT injections in mice, monkeys and humans [22–24]. The safety of adenoviral vectors in the human eye has been demonstrated in two Phase I clinical trials; one in a pediatric (1 to 7 years old) population and the other in elderly (62 to 97 years old) patients [25,26]. In GenVec’s first Phase I study, 1 IVT injection of a replication-deficient, serotype 5, adenoviral (Ad5) vector into the eyes of elderly patients with severe wet AMD resulted in only mild and transient inflammation in some patients. This was not dose-dependent, and a maximum tolerated dose was not reached even at the highest tested dose of 1×10^9.5^ particle units (pu). Likewise, in children with retinoblastoma, an Ad5-based vector containing thymidine kinase was well tolerated at doses of 1×10^11^ pu with up to a total of 5 individual injections. Thus, in humans, adenoviral vectors given intravitreally resulted in less of an immune response than originally anticipated [27].

**Figure 1:**
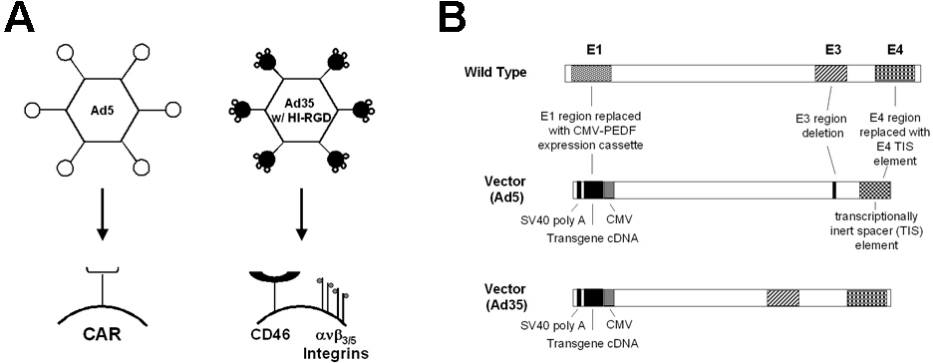
Schematic representation of Adenoviral vectors. **A:** This is a schematic diagram illustrating the cellular receptors for Ad5 and Ad35-based vectors with Ad35 vector containing the RGD motif inserted in the HI loop of the fiber knob. **B:** This is a schematic representation of wild-type adenovirus and an adenovector demonstrating the E1, E4 and partial E3 deletion (Ad5), or E1 only deletions (Ad35) with a SV40 poly A stop sequence, transgene (GFP, Luciferase, or PEDF) cDNA, and a human CMV promoter.

Ad5-based vectors have been traditionally used in pre-clinical experiments and clinical trials reported throughout the literature [28–34]. Ad5 vectors mediate their attachment and entry into cells using the Coxsackie and Adenovirus Receptor (CAR) [35–39]. In contrast, Ad35 vectors do not interact with CAR, but instead bind to CD46 receptors (also known as MCP-membrane cofactor protein) [40–42]. In addition, one can incorporate into the knob portion of the vector the sequence arginine-glycine-aspartic acid (cyclic RGD). This modification promotes vector binding to α_v_β_3/5_ integrin receptors that can function as secondary Ad35 receptors [43]. Figure 1A is a diagram of the wild-type Ad5 capsid and its endogenous receptor, CAR, and the Ad35 vector with its native tropism for CD46 sites on cells and the RGD modification that enhances binding to cell surface α_v_β_3/5_ integrin receptors. Several groups have shown that the majority of humans do not have circulating neutralizing antibodies for Ad35 vectors [44,45]. Since neutralizing antibodies are reduced for Ad35 compared to Ad5 vectors, we hypothesize that host immune response to Ad35 vector may be reduced and thereby may contribute to prolonged gene expression with an Ad35 vector. In summary, we have discovered an alternate delivery system that may improve transgene expression kinetics of a therapeutic protein following a single intraocular injection, thereby decreasing the number of intraocular injections, and providing improved safety and efficacy for AMD treatment compared to Ad5 vectors.

## Methods

### Animals

Female, C57BL/6 mice (6–8 weeks old; Harlan Laboratories, Chicago, IL) were acclimated for approximately 1 week before use. The animals were housed under controlled lighting conditions (12 h:12 h light-dark), and were given food and water ad libitum during the experiments. All experiments were conducted in accordance with the Association for Research in Vision and Ophthalmology (ARVO) statement for the Use of Animals in Ophthalmic and Vision Research and the guidelines established by the Institutional Animal Care and Use Committee (IACUC) at GenVec, Inc.

### Adenoviral vectors expressing GFP, luciferase, or PEDF

Production and quantification of type-5 (Ad5) and type-35 (Ad35) adenoviral vectors expressing green fluorescent protein (GFP), luciferase and human pigment epithelium-derived factor (PEDF) from a cytomegalovirus (CMV) immediate early promoter expression cassette has been previously described [46–51]. Both the Ad5 and Ad35 vectors expressing GFP, luciferase or PEDF were constructed using GenVec's AdFAST technology, which employs homologous recombination methods in *Escherichia coli* to quickly restructure the adenoviral vector genome. The human cytomegalovirus (CMV) promoter was cloned into the deleted E1 region of the adenovirus shuttle vector, upstream of the transgenes and SV40 poly A sequences. The expression cassettes are oriented such that the transgenes are transcribed from right to left relative to the adenovirus genome. During construction, a RGD-4C peptide was incorporated into the HI loop of the Ad fiber knob as previously described [52–54]. Ad5 vectors were devoid of the E1, E3, and E4 adenovirus replication regions, whereas Ad35 vectors were devoid of the E1 region (Figure 1B). Adenovirus particles were purified using 3 successive rounds of cesium chloride (CsCl) gradient centrifugation. The vector was formulated for storage and was assayed for potency, purity, sterility, and absence of replication competent adenovirus (RCA).

### Intravitreal injection of adenoviral vectors

IVT injections of vector were performed with pulled-borosilicate glass micropipettes (World Precision Instruments, Sarasota, Florida) using a pump microinjection apparatus (Harvard Apparatus, Holliston, MA), as previously described [55]. Each calibrated micropipette delivered approximately 2 µl of buffer containing the specified number of viral particle units (1×10^9^ pu) upon depression of a foot switch. Mice were anesthetized with a 100 µl intraperitoneal injection of ketamine hydrochloride (40 mg/kg) and xylazine (12 mg/kg), both from The Butler Company (Columbus, OH). For each mouse eye, one drop of 0.5% proparacaine hydrochloride (Bausch & Lomb, Tampa, FL) was administered as a topical anesthetic. Under a dissecting microscope (Nikon, Melville, NY), the sharpened tip of the micropipette was passed through the sclera, just behind the limbus into the vitreous cavity. The foot switch was depressed, which caused a jet injection of the vector to penetrate the vitreous space. At specified time points, eyes were observed for in vivo GFP expression or mice were humanely euthanized by asphyxiation with carbon dioxide followed by cervical dislocation, eyes excised and analyzed for luciferase activity or PEDF levels.

### In vivo detection of GFP expression by fluorescence microscopy

GFP expression was visualized in the anterior segment of the eye on Days 1, 7, 14, 28, 60, 90, and 120 following one IVT injection of Ad5 or Ad35.GFP based vectors using a fluorescence stereomicroscope (Leica Microsystems, Wetzlar, Germany). At each time point, mice were anesthetized with a 100 µl intraperitoneal injection of ketamine hydrochloride (40 mg/kg) and xylazine (12 mg/kg), both from the Butler Company (Columbus, OH); and GFP expression observed noninvasively by placing the animal under a microscope and observing the anterior, exterior ocular surface of the mouse eye. Representative microscopic images of GFP expression in the epithelial layer of the cornea was captured by viewing the anterior surface of the eye using the Leica MZFLIII stereomicroscope with a vertical fluorescence illuminator equipped with filters for GFP, and a SPOT RT slider color digital camera (Diagnostic Instruments, Inc., Sterling Heights, MI). Images were obtained using a standardized exposure time.

### Assessment of luciferase activity and PEDF levels

To assess the time course of luciferase activity and PEDF levels following 1 IVT injection, 1×10^9^ pu were injected, and eyes were enucleated at specified time points and analyzed for luciferase activity or PEDF levels. Immediately following enucleation, eyes were snap frozen in dry ice and stored at –80 °C. A pre-cooled mortar and pestle on dry ice with liquid nitrogen provided mechanical homogenization of the eyes.

For luciferase expression, homogenized eyes were lysed with 300 µl 1X Reporter Lysis Buffer (Promega, Madison, WI). Resultant lysates were analyzed with a luciferase assay system according to the manufacturer’s protocol (Promega). For PEDF levels, homogenized eyes were lysed with 100 µl 0.1% Triton X-100 (Sigma Aldrich, St. Louis, MO) in sterile filtered Dulbecco's Phosphate Buffered Saline (1X PBS) without calcium or magnesium (Cambrex Corporation, East Rutherford, NJ), and PEDF levels were assessed using a sandwich enzyme-linked immunoabsorbant assay (ELISA) developed by GenVec, Inc. [56–58]. The total protein concentration was determined to normalize the measurement of luciferase activity and PEDF levels based on a Bradford dye binding procedure with a protein assay (Bio-Rad, Hercules, CA).

### Assessment of adenoviral genomic DNA by polymerase chain reaction

Viral genomic DNA was extracted from whole eye using the DNeasy Tissue Kit (Qiagen, Valencia, CA) according to the manufacturer’s instructions. PCR was performed using TaqMan 2X universal master mix (Applied Biosystems, Foster City, CA). Final concentrations of primers and probe were: 200 nM of each primer, 100 nM of the probe, 100 ng of template DNA, and nuclease-free water in a total volume of 50 µl per well using the ABI Prism 7700 Sequence Detection System and 7700 SDS Software (Applied Biosystems). Nuclease-free water was used as a non-template negative control. The primers and probe sets were designed from the pIX gene by Applied Biosystems. The sequences were as follows: forward primer, 5′-CGC GGG ATT GTG ACT GAC T-3′; reverse primer, 5′-GCC AAA AGA GCC GTC AAC TT-3′; fluorogenic detection probe, 5′-FAM-AGC AGT GCA GCT TCC CGT TCA TCC-TAMRA-3′. The reactions were thermal cycled using the following conditions: 50 °C for 2 min, 95 °C for 10 min, followed by 40 cycles of 95 °C for 15 s, and 60 °C for 1 min. The data were processed using the instrument’s sequence detection software package.

### Mouse model of laser-induced choroidal neovascularization

Approximately 28 days following 1 IVT injection of Ad5 or Ad35-based vectors as previously described, animals were anesthetized and pupils dilated with Accutome^®^ (1% Accu-tropicamide; Bausch & Lomb). A drop of 0.5% proparacaine hydrochloride (Bausch & Lomb) was administered as a topical anesthetic and a drop of Goniosol^®^ (2.5% hydroxypropyl methylcellulose; CibaVision Ophthalmics, Atlanta, GA) to facilitate viewing and focusing of the laser beam. Diode laser photocoagulation was used to rupture the Bruch’s membrane at 3–4 locations in both the right and the left eyes. Laser photocoagulation was performed around the optic disc at a wavelength of 532 nm (75 μm spot size; 0.1 s duration, 120 mV) using a slit lamp delivery system (Oculight GLx; Iridex, Mountain View, CA) and a hand-held coverslip (VWR, West Chester, PA) as a contact lens. Burns were performed at the 3, 6, 9, and 12 o' clock positions; approximately 2–3 disc diameters from the optic nerve so that each burn could be identified postmortem. Production of a vaporization bubble at the time of laser indicated rupture of the Bruch's membrane that is an important factor in obtaining choroidal neovascularization (CNV) [59]. After 14 days, mice were euthanized and the amount of CNV quantitated as previously described [60,61].

### Measurement of laser-induced choroidal neovascularization

Two weeks following laser treatment, CNV lesion size was measured in choroidal flat mounts [62]. Mice used for the flat mount technique were anesthetized and perfused with 1.0 ml PBS containing 50 mg/ml fluorescein-labeled dextran (2×10^6^ average MW, Sigma) as previously described [62,63]. Eyes were harvested and fixed overnight in 10% phosphate-buffered formalin (VWR). The cornea and lens were removed and the entire retina carefully dissected from the eyecup. Radial cuts (4–7) were made from the edge to the equator and the eyecup flat mounted to a glass microscope slide (VWR) using Vectashield^®^ mounting medium (Vector Laboratories, Inc. Burlingame, CA) with the sclera facing down. Flat mounts were examined by fluorescence microscopy using a fluorescence stereomicroscope (Leica Microsystems) and images captured using a SPOT RT digital camera (Diagnostic Instruments, Inc.). Image-Pro Plus software (Media Cybernetics, Silver Spring, MD) was used to measure the total area of each burn corresponding with a fibrovascular scar. The areas within each eye were averaged to obtain one value, and the means were calculated for each treatment group.

### Statistical analysis

Data are expressed as the mean±SEM. An overall test for treatment effect was first performed with one-way ANOVA (ANOVA). If the overall test indicated a significant treatment effect, individual treatments were compared with the vector-only treatment groups, using Bonferroni analyses, which adjusted for multiple comparisons. The level established for statistical significance was p<0.05 (two-tailed Student *t*-test) [64]. All analyses were performed using OriginPro 7.5 software (Origin Laboratories, Northampton, MA).

## Results

### Ad35.GFP vector results in prolonged GFP expression

**Figure 2:**
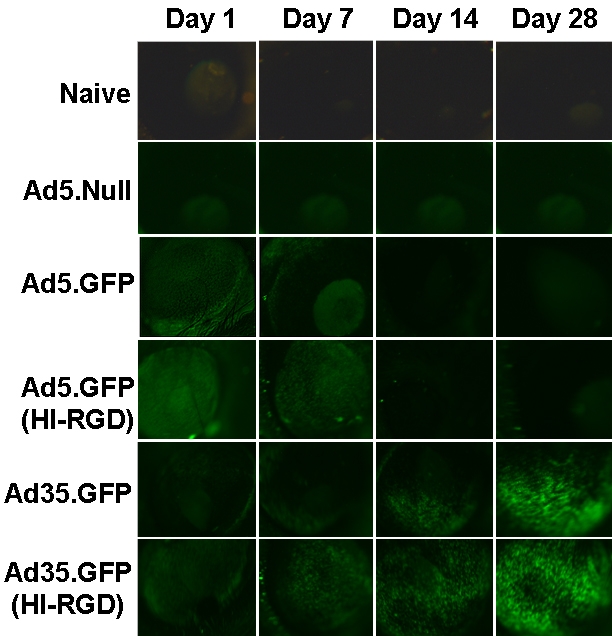
GFP expression following Ad35-based vector results in longer transgene levels. The anterior segment of whole mouse eyes was examined for GFP expression following a single IVT injection of 1×10^9^ pu of either Ad5.Null, Ad5.GFP (±HI-RGD), or Ad35.GFP (±HI-RGD). Naïve mice served as negative controls. Data are shown as GFP expression on Days 1, 7, 14, and 28 post IVT injection. Data are representative photographs of 1 mouse from each treatment group (n=15 mice/treatment group).

**Figure 3:**
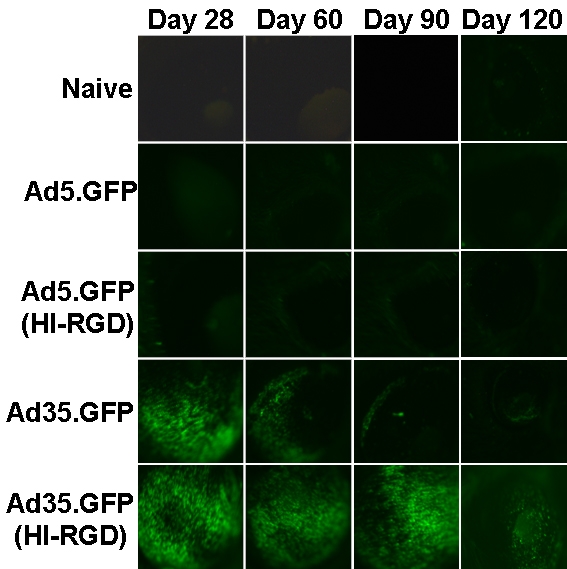
GFP expression following Ad35-based vector results in longer transgene levels. GFP expression in the anterior segment of whole mouse eyes following 1 IVT injection of 1×10^9^ pu of either Ad5.GFP (±HI-RGD) or Ad35.GFP (±HI-RGD). Naïve mice served as negative controls. Data are shown as GFP expression on Days 28, 60, 90, and 120 post IVT injection. Data are representative photographs of 1 mouse from each treatment group (n=5 mice/treatment group).

To assess the gene expression profile from Ad35-based vectors, we observed GFP expression in the eye on Days 1, 7, 14, and 28 post vector injection. Mice received 1 IVT injection of a vector without a marker gene (Ad5.Null), Ad5.GFP, or Ad35.GFP (**±**HI-RGD motif). Figure 2 shows representative images of the surface of the anterior cornea of a mouse eye over time. In naïve mice and those injected with Ad5.Null (empty cassette), green fluorescent cells were absent. However, eyes that received Ad5.GFP (**±**HI-RGD motif) showed an induction in GFP signal within 1 day post-injection. Subsequent to Day 7, GFP expression rapidly declined in eyes that received Ad5.GFP (**±**HI-RGD motif). In contrast, the response with the Ad35-based vectors (±HI-RGD motif) resulted in minimal GFP signal on Day 1, which gradually increased with time. By Day 28 the signal exceeded that observed on Day 1. GFP expression in eyes given Ad5.GFP with HI-RGD was identical to Ad5.GFP alone. Thus, the HI-RGD motif is not the reason for the altered GFP expression profile observed with the Ad35 vectors. Based on these initial findings, we continued to monitor the GFP signal in the animals. The GFP signal continued beyond 4 months in the Ad35.GFP (HI-RGD) treated eyes (Figure 3), but diminished to background levels by 8 months (data not shown).

### Adenovector genomes are stable in the eye

**Figure 4:**
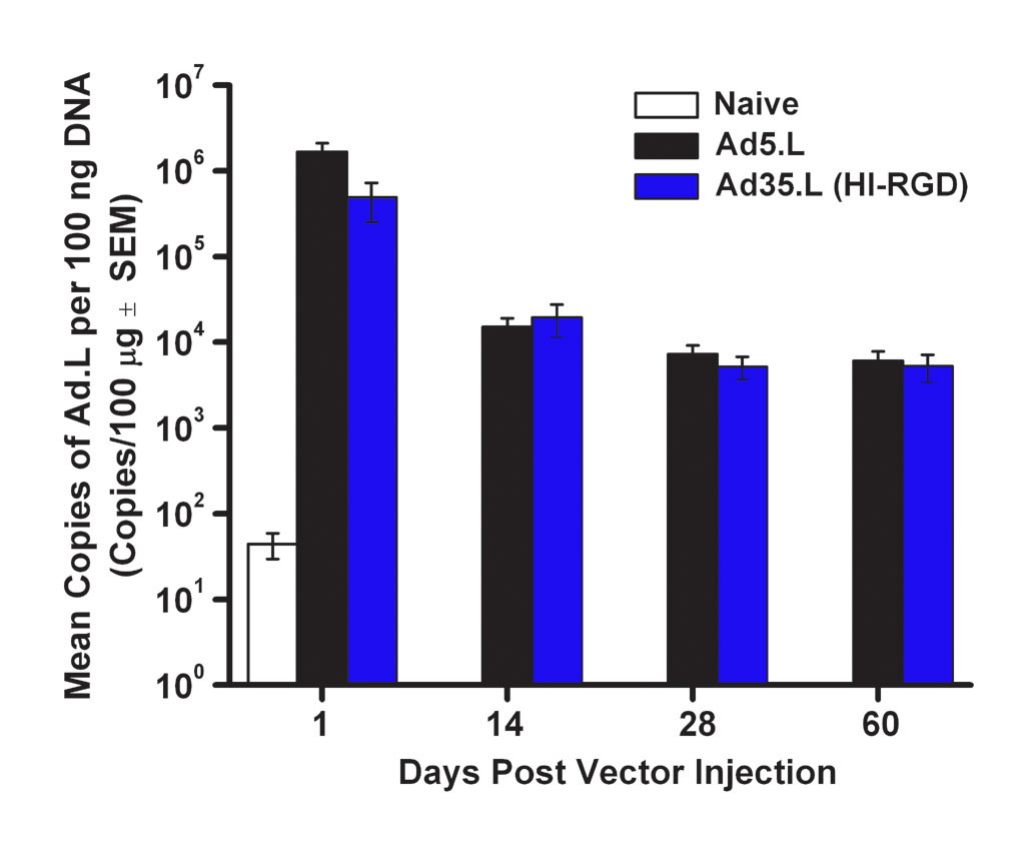
Quantitative analysis of adenovector genomes by polymerase chain reaction. Adenovector genomes in whole mouse eyes following 1 IVT injection of 1×10^9^ pu of either Ad5.L or Ad35.L (HI-RGD). On Days 1, 14, 28, and 60 post vector injection, eyes were harvested and adenovector genomes quantitated using qPCR. Data are expressed as the mean±SEM (error bars) with an n=5 mice/treatment group/time point.

Based on the prolonged GFP expression profile with Ad35.GFP or Ad35.GFP (HI-RGD), we wanted to verify that the genomes or DNA from the Ad35 vector backbone were still present in the mouse eye. For these studies, we selected Ad35.L (HI-RGD) since there was no difference between the construct ±HI-RGD. Mice received 1 IVT injection of Ad5.L or Ad35.L (HI-RGD; Figure 4). Animals were euthanized and eyes harvested on Days 1, 14, 28, and 60. Quantitative analysis of the adenovector levels showed an initial loss of vector genome on Day 1. Vector levels are comparable following Ad5 or Ad35-based vector delivery with a 2-log decrease from Day 1 to Day 14. Stability was observed, as there was no change in levels out to Day 60 post-vector injection. An additional study showed that genomes were still present up to 1 year following 1 IVT of Ad5 vector [65].

### Ad35.L vector results in prolonged luciferase activity

**Figure 5:**
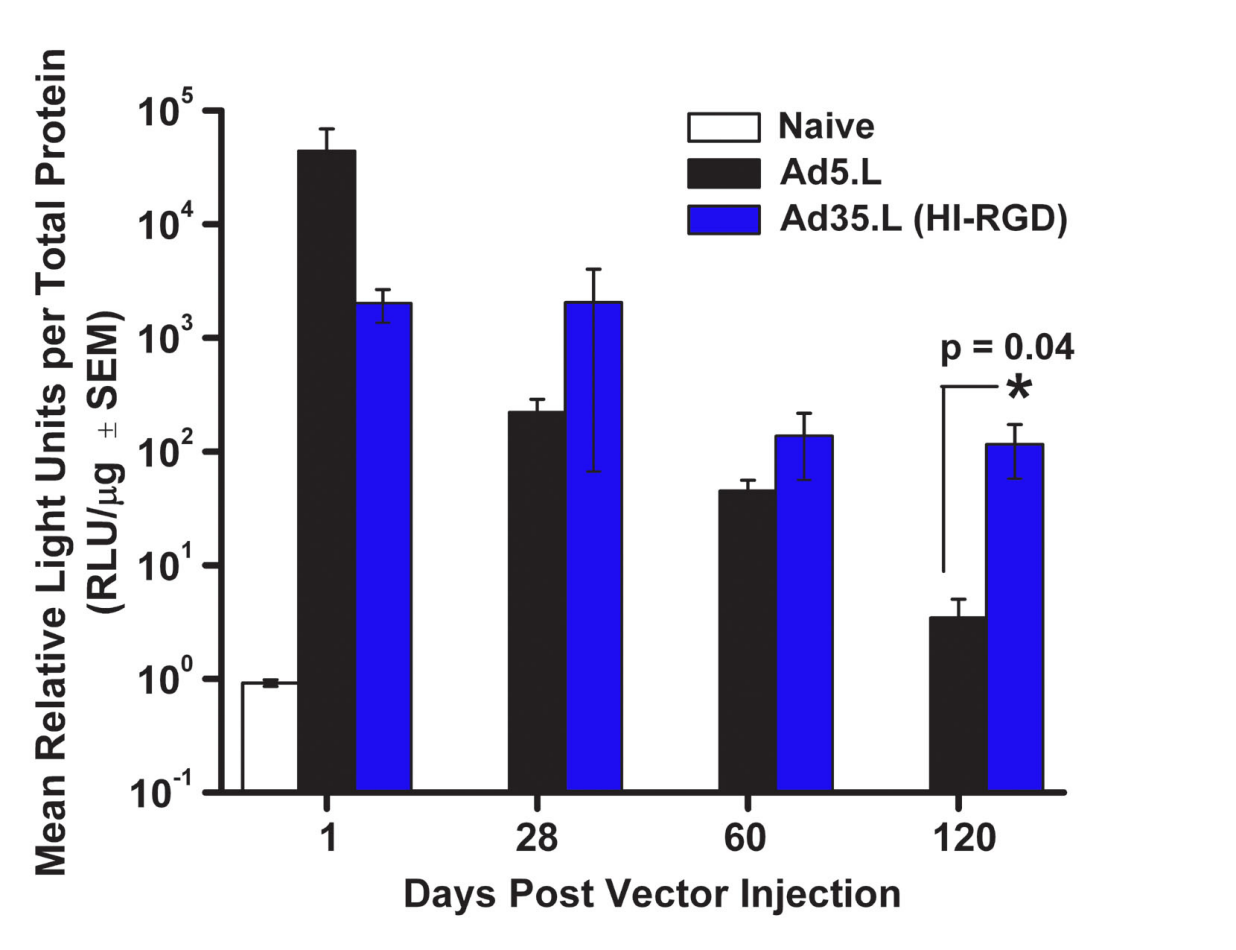
Luciferase expression profile following 1 IVT injection of Ad5- or Ad35-based vectors. Luciferase activity in mouse eyes following a single IVT injection of 1×10^9^ pu of either Ad5.L or Ad35.L (HI-RGD). Naive animals served as negative controls. On Days 1, 28, 60, and 120 post vector injection, eyes were harvested and luciferase activity assessed. Data are expressed as the mean±SEM (error bars) with an n=15 mice/treatment group/time point, except for Day 120, which has an n=5 mice/treatment group. The asterisk indicates a statistically significant difference between the Ad5 and Ad35 groups on Day 120 post injection (p<0.05, two-tailed Student’s *t*-test).

To provide quantitative confirmation of the GFP expression profile, we examined luciferase activity following 1 IVT injection of either Ad5.L or Ad35.L (HI-RGD; Figure 5). Luciferase activity was initially high at Day 1 with a rapid decline out to Day 120 following administration of Ad5-based vector. Luciferase activity was approximately 2-logs lower at Day 1 in Ad35 (HI-RGD) compared to Ad5-treated eyes. However, luciferase activity remained relatively stable from Day 1 to Day 120 following 1 IVT injection of Ad35 (HI-RGD). These results indicate that a single IVT injection of Ad35.L (HI-RGD) can result in higher and prolonged luciferase activity out to at least 120 days as compared to Ad5.L.

### Alternate serotype results in high therapeutic transgene levels

**Figure 6:**
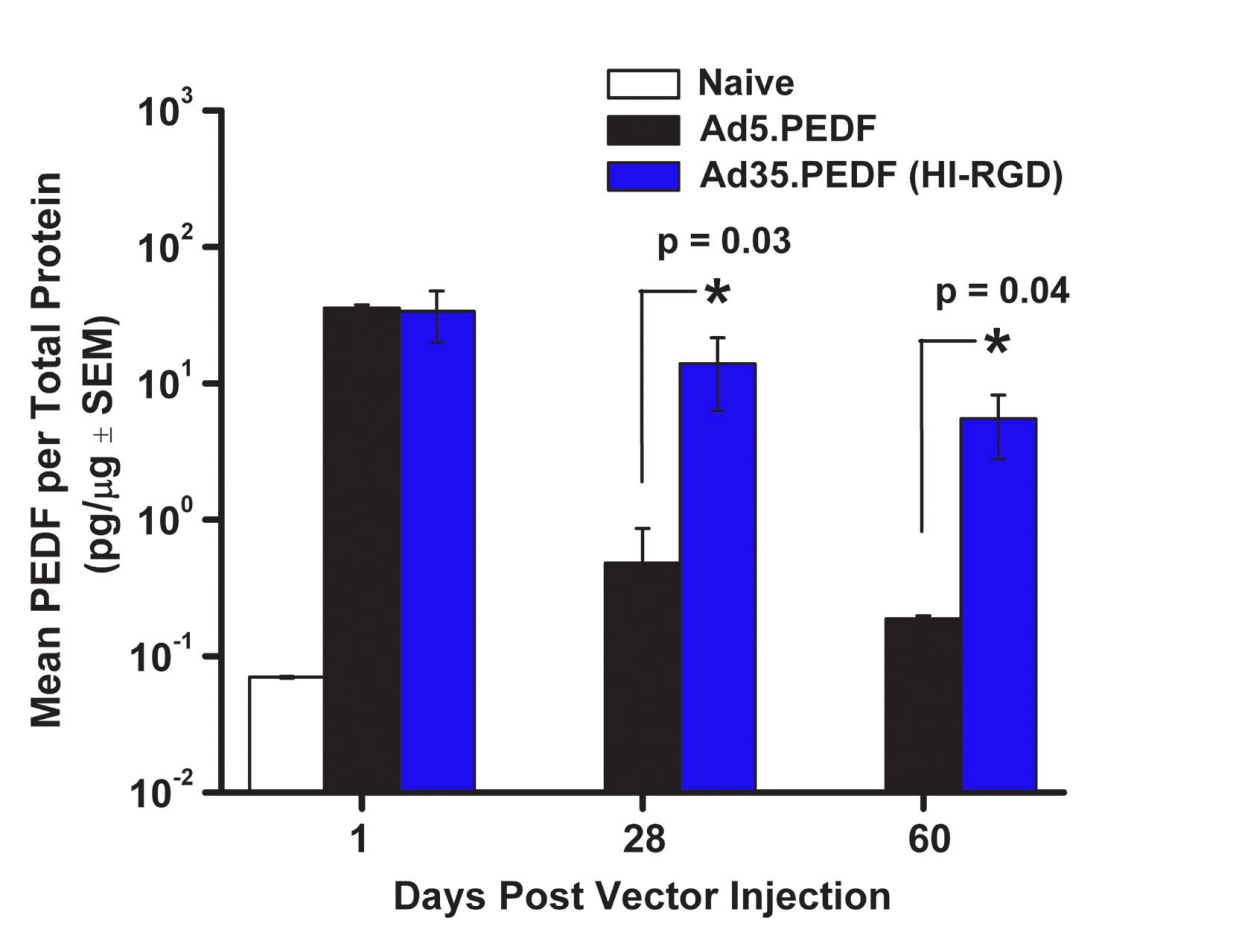
PEDF expression profile following 1 IVT injection of Ad5 or Ad35-based vectors. PEDF levels in mouse eyes following a single IVT injection of 1×10^9^ pu of Ad5.PEDF or Ad35.PEDF (HI-RGD). Naive animals served as negative controls. On Days 1, 28, and 60 post vector injection, eyes were harvested and PEDF levels quantitated by ELISA developed by GenVec, Inc. Data are expressed as the mean±SEM (error bars) with an n=15 mice/treatment group/time point, except for Day 60 which has an n=5 mice/treatment group. The asterisk points to a statistically significant difference between the Ad5 and Ad35 groups on Day 28 and 60 post injection (p<0.05, two-tailed Student’s *t*-test).

To confirm the marker gene data, we conducted similar experiments using Ad5.PEDF and Ad35.PEDF (HI-RGD) administered once intravitreally (Figure 6). Human PEDF levels were measured using a sensitive PEDF ELISA as previously defined [60]. This PEDF ELISA does not detect mouse PEDF and has a sensitivity level of approximately 0.07 ng/ml. Interestingly, we found in this study that PEDF levels were comparable between Ad5.PEDF and Ad35.PEDF (HI-RGD) within 1 day post injection. However, by Day 60, PEDF levels had dropped by more than 1 log for Ad5.PEDF- treated eyes whereas in Ad35.PEDF (HI-RGD) treated animals, the PEDF levels were approximately 5–14 pg/μg protein. Based on estimations by Stellmach et al. [66,], Raisler et al. [67], and Mori et al. [61] scant levels of PEDF are sufficient to inhibit blood vessel growth (on the order of approximately 1–2 pg/μg protein or 1–2 ng/mg protein). These results indicate that 1 IVT injection of Ad35.PEDF (HI-RGD) can result in elevated PEDF levels out to at least 60 days.

### Ad35.PEDF vector inhibits choroidal neovascularization

**Figure 7:**
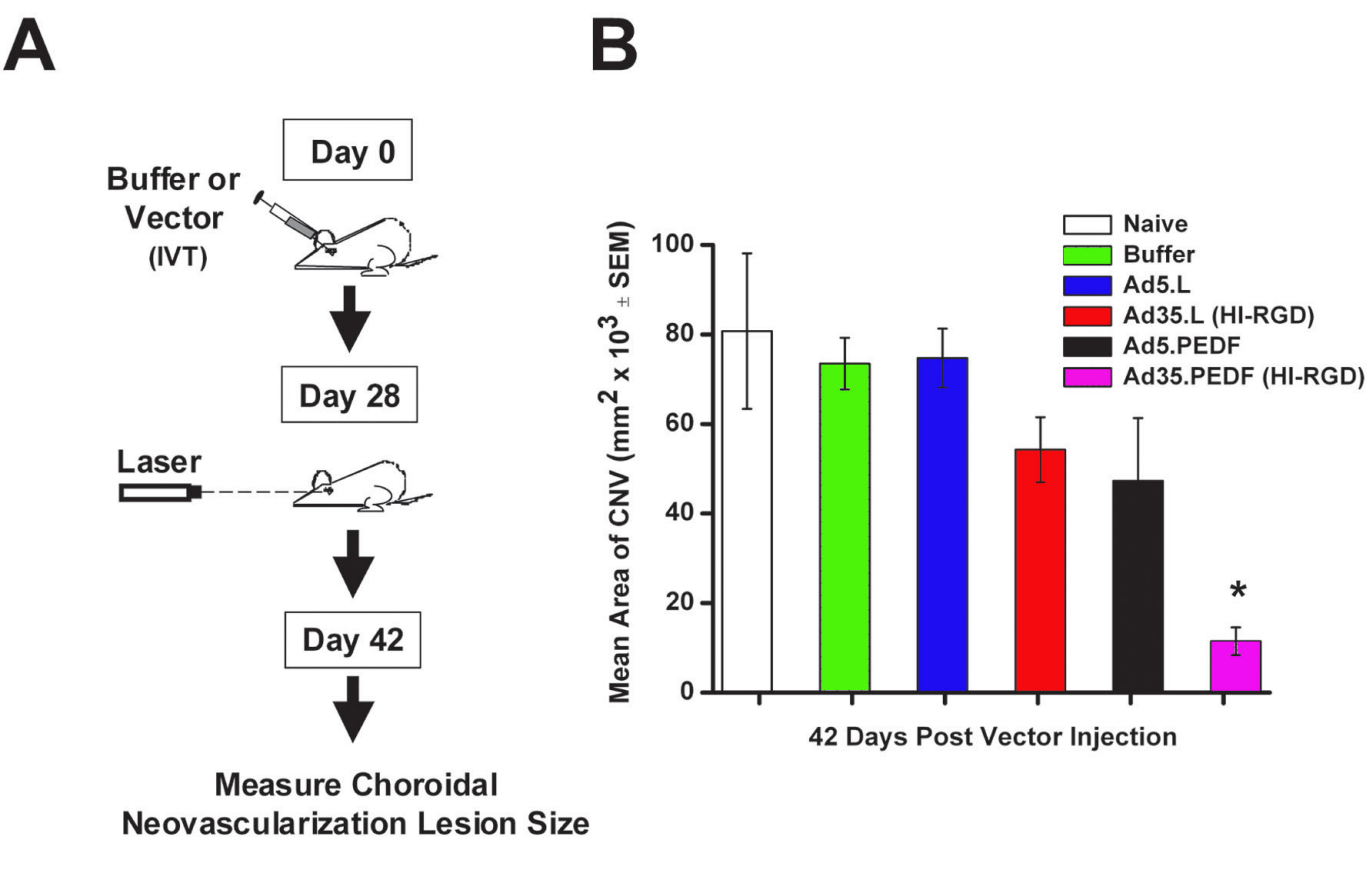
In vivo biologic activity of Ad35-based vector in the mouse laser-induced CNV model. **A:** This is a schematic illustration of the mouse laser-induced CNV model. **B:** Mice were untreated (naïve) or received 1 IVT injection (1×10^9^ pu) of either Ad5.L, Ad35.L (HI-RGD), Ad5.PEDF, or Ad35.PEDF (HI-RGD). Approximately 28 days following buffer or vector treatment, laser induction of CNV was performed. Fourteen days following laser induction, mice were perfused with fluorescein-labeled dextran, eyes harvested, choroidal flat mounts prepared, and CNV lesion size quantitated. Data are expressed as the mean±SEM (error bars) with an n=6 mice/treatment group, except for Ad5.L and Ad35.L (HI-RGD), which had an n=5 mice/treatment group. The asterisk points to a statistically significant difference between the Ad35.PEDF (HI-RGD)-treated group and all other treatment groups on Day 42 post injection (p<0.01, two-tailed Student’s *t*-test).

The mouse model of laser-induced CNV, shown in Figure 7A, mimics several features of wet AMD in humans [49,62]. This in vivo model is commonly used to screen potential anti-angiogenic compounds for their ability to inhibit CNV. To assess the ability of an alternate serotype vector to inhibit abnormal blood vessel growth in the eye, animals received no injection (naïve) or a single, IVT injection of buffer, Ad5.L, Ad35.L (HI-RGD), Ad5.PEDF, or Ad35.PEDF (HI-RGD) on Day 0. Twenty-eight days later, experimental CNV was induced by creating a laser burn that would disrupt Bruch’s membrane of the mouse retina. Fourteen days later, the mice were euthanized, and choroidal flat mounts were prepared to quantitate lesion size (Figure 7B). Based on these data, PEDF expression from an Ad35.PEDF (HI-RGD) vector was able to inhibit CNV lesion growth by greater than 80% at Day 42 as compared to the no-injection control (Figure 7B).

## Discussion

We, and others, have investigated the utility of Ad35 alternate vectors for possible therapeutic development. Ad35 provides advantages over the Ad5 vectors with respect to durability of transgene expression, level of transgene expression and perhaps a superior safety profile compared to Ad5 vectors [44,45,68].

Although Ad5 vectors have shown good safety in numerous clinical trials in areas including cardiology [69,70], oncology [71–73], and ophthalmology [25,26,74], there continues to be a quest to improve beyond the Ad5 class of vectors. One potential limitation of the Ad5 vector platform is that this adenovector serotype traditionally does not provide long-term expression. The transient expression is thought to be due to various immune responses to the vector [75–78]. We report here that an Ad35 alternate vector can result in up to 4 months of GFP expression in eyes, and diminishing to background levels by 8 months, following 1 IVT injection. Interestingly, Mallam et al. [68] also found 8-month GFP expression in animals given 1 subretinal injection of an Ad5 vector with an Ad35 knob. Mallam et al. [68] also noted that GFP expression was higher in cells transduced with an Ad35 knob vector versus a vector with an Ad5 knob as determined by Fluorescence Activated Cell Sorting (FACS) analyses.

Furthermore, the observation of strong GFP expression following 1 IVT injection of Ad35.GFP (**±**HI-RGD) into the eyes of mice was a reasonable finding since it has been reported that in mice tissues, the testes and the eye express CD46 [68,79–82]. In humans, CD46 is present in the corneal epithelium and photoreceptor cells [83]. In mice, Mallam et al. found that 1 subretinal injection with an Ad35 knob vector containing GFP resulted in green fluorescent cells within the photoreceptor inner and outer segments and the retinal pigment epithelial cells of the mouse eye [68]. Our results suggest that following 1 IVT injection into a mouse eye with an Ad35 vector, cells of the anterior segment are transduced. However, the precise cells, which are infected remains unknown.

Although the transgene expression profiles differed between the two marker genes, GFP and luciferase, compared to the secreted human PEDF protein, our data show that an Ad35 vector administered intravitreally to the eye results consistently in higher levels of each transgene at later time points (e.g., Days 60 and 120) than the Ad5 serotype. In the case of the anti-angiogenic protein, human PEDF, these levels were higher than the predicted therapeutic levels (1–2 pg/μg) [61,66,67], reported to have biologic activity (i.e., inhibit abnormal blood vessel growth) at a later time point (Day 60). The difference in transgene expression profiles could be attributed to variability in protein turnover of the different proteins in transduced cells. Our data (Figure 7B) support this valued finding that only a low amount of PEDF is enough to block neovascularization in the eye. Although the Ad35 backbone also seems to have some antivasculature effect (null effect), major effect is attributable to the presence of PEDF [49,55,56,60,84].

Our data demonstrate the feasibility and utility of Ad35 vectors as a delivery mode for medical applications in the eye without frequent repeat intraocular injections. Moreover, the ability to achieve long-term gene expression in the eye provides a possible therapeutic benefit of Ad35 over Ad5 vectors for the treatment of chronic ocular diseases.
